# 人大细胞肺癌干细胞样细胞的富集及其功能研究

**DOI:** 10.3779/j.issn.1009-3419.2011.06.19

**Published:** 2011-06-20

**Authors:** 文科 月, 锋 焦, 彬 刘, 嘉琮 尤, 清华 周

**Affiliations:** 300052 天津，天津医科大学总医院天津市肺癌研究所，天津市肺癌转移与肿瘤微环境重点实验室 Tianjin Key Laboratory of Lung Cancer Metastasis and Tumor Microenvironment, Tianjin Lung Cancer Institute, Tianjin Medical University General Hospital, Tianjin 300052, China

**Keywords:** 肺肿瘤, 肺癌干细胞, 无血清悬浮培养, 肺癌球体细胞, Lung neoplasms, Lung cancer stem cell, Serum-free suspension, Spheroid lung cancer cell

## Abstract

**背景与目的:**

目前国内外还没有确切的、得到公认的肺癌干细胞的筛选标记分子、指标和方法，常用方法为通过流式细胞技术，借鉴其他肿瘤干细胞分选标记来分选肺癌干细胞，但其筛选特异性低、工作量巨大。本研究采用无血清悬浮培养法富集肺癌干细胞，对肺癌干细胞的筛选方法进行探索。

**方法:**

采用添加生长因子的无血清培养基对人大细胞肺癌细胞株L9981进行悬浮培养，获得肺癌细胞球体。对含血清培养贴壁L9981细胞和无血清培养成球后的L9981细胞，通过显微镜下观察比较二者的生物学形态，应用Vi-cell细胞活力分析仪计数细胞并绘制生长曲线比较二者的增殖能力，通过Transwell实验研究它们的侵袭能力差异，并通过接种裸鼠观察二者在体内的成瘤性来研究肺癌细胞球体的生物学功能。

**结果:**

与血清贴壁培养L9981细胞相比，无血清培养L9981细胞成球形生长，贴壁L9981和L9981球体细胞的倍增时间分别为（56.05±1.95）h和（33.00±1.44）h，球体细胞的侵袭和成瘤能力分别为贴壁L9981细胞的5倍和20倍。

**结论:**

通过无血清悬浮培养的L9981细胞可以形成肺癌球体细胞群，L9981球体细胞的侵袭和成瘤能力均明显高于贴壁L9981细胞，显示L9981球体细胞中富集了肺癌干细胞样的细胞。无血清悬浮培养肺癌球体细胞可作为富集肺癌干细胞样细胞的一种候选方法。

## Abstract (en)

**Background and objective:**

There are no universal method to recognize and screen for lung cancer stem cell markers and indicators. Commonly used methods are flow Cytometry and learning from other cancer stem cell sorting tags to sort lung cancer stem cells. But this method has low specificity screening, the workload is huge. In this study, Serum-free suspension culture was used to enrich lung cancer stem cells, and explore method for lung cancer stem cell screening.

**Methods:**

Human large lung cancer cell line-L9981 was cultured in serum-free and growth factors added medium, and spheres were obtained. Then the morphological differences of sphere cells and adherent L9981 cells cultured in serum-containing mediums are observed. Cell proliferation was analyzed by Vi-cell viability analyzer; invasion ability was tested by transwell assay; and *in vivo* tumorigenicity of the two groups of cells was studied in nude mouse.

**Results:**

Compared with adherent L9981 cells cultured in serum-containing mediums, cells cultured in serum-free medium display sphere appearance. Doubling time of adherent cells and sphere cells are (56.05±1.95) h and (33.00±1.44) h respectively; Spheroid cells had higher invasion and tumorigenicity ability, 5 times and 20 times respectively, than adherent cells.

**Conclusion:**

Suspension cultured L9981 in Serum-free medium could form spheroid populations. Cells in spheres had higher ability of invasion and Tumorigenicity than adherent L9981 cells. These results indicated spheroid L9981 cells contained enriched lung cancer stem cells, and Serum-free suspension culture can be a candidate method for enriching lung cancer stem cell.

近来有研究和干细胞理论表明，包括肺癌在内的肿瘤被认为可能是是一种干细胞疾病。目前肺癌干细胞研究的焦点为肺癌干细胞的分离、纯化和鉴定，但肺癌干细胞表面标记物还不明确，阻碍了肺癌干细胞的研究。借鉴其他肿瘤干细胞分选标记来分选肺癌干细胞筛选特异性低、工作量巨大。不同病理类型的肺癌可能起源于不同的肺癌干细胞，对肺组织细胞分化的阶梯层次尚不完全清楚^[1-5]^。这进一步增加了肺癌干细胞筛选的难度。明确能够有效分离和鉴定肺癌干细胞的表面标记是肺癌干细胞研究的重要前提。2008年Eramo^[6]^等提出CD133是一个小细胞肺癌（small cell lung cancer, SCLC）和非小细胞肺癌（non-small cell lung cancer, NSCLC）中都存在的有效的肺癌干细胞分选标记物，但对此标记存在较大争议且新的分选标记层出不穷但都未有定论。

本研究应用无血清悬浮培养成球技术从肺癌细胞株中富集肺癌干细胞群，并对其进行生物学功能研究，对建立有效分离肺癌干细胞的方法进行探索，为后续进一步明确肺癌干细胞标记奠定实验基础。

## 1 材料与方法

### 1.1 材料

#### 1.1.1 细胞株

人大细胞肺癌细胞株L9981来自天津总医院天津市肺癌研究所。

#### 1.1.2 试剂与材料

DMEM/F12培养基（Gibico公司）；小牛血清（Gibico公司）；0.25%胰酶（Gibico公司）；牛血清白蛋白（BSA）；PeproTeth公司提供的上皮生长因子（Epidermal growth factor, EGF）、人胰岛素样生长因子-1（Insulin-like growth factor-1, IGF-1）、小鼠碱性成纤维生长因子（Basic fbroblast growth factor, bFGF）；DMEM/F12培养基中的需添加的SIGMA公司的提供的配方成分：右旋葡萄糖（G7021）、人转铁蛋白（TI147）、亚硒酸钠（55261）、牛血清白蛋白（BSA）（A2153）、孕酮（P8783）、牛胰岛素（10516）、腐胺（P5780）、肝素（H3149）和青链霉素；actuates酶（Gibico公司）；mate-riel（Becton Dickinson公司）；超低吸附培养皿和六孔板（Corning公司）；FACS Asia流式细胞仪（BD公司）；Vi-cell细胞活力分析仪（Beckman coulter公司）。

### 1.2 实验方法

#### 1.2.1 无血清悬浮培养细胞

按照Eramo等^[6]^的无血清成球培养法，用无血清DMEM/F12培养基将L9981细胞调整至2.0×10^4^/mL，取10 mL细胞悬液接种于超低吸附培养皿中，其中每毫升培养液液中加入20 ng/mL IGF-1，20 ng/ mL EGF和10 ng/mL bFGF各20 μL。每隔2天-3天添加一次生长因子，并进行半量换液，直至球体形成。收集成球最高时期的细胞，离心后应用actuates酶将细胞球体消化为单细胞悬液，再次以上述密度传代。重复上述培养过程，直至能够稳定传代培养。在倒置显微镜下观察细胞成球的过程和细胞球形态。

#### 1.2.2 检测肺癌L9981成球细胞的增殖能力

取无血清悬浮培养成球后的肺癌球体细胞，调整细胞悬液浓度为1×10^5^/mL，采用含10%血清的DMEM/F12培养基在6孔板培养细胞。每个孔加入2 mL，共设10个点，每24 h计数一个点，每个点设3个重复。每24 h收集细胞消化后用Vi-cell细胞活力分析仪计数一个点。对照组为相同浓度的含血清贴壁培养的L9981细胞。以培养时间为横轴，以相对应时间测得的细胞数为纵轴绘制生长曲线，并根据如下公式计算细胞在对数生长期的倍增时间。Td=t×log2/log（Nt/N0），t代表对数生长期时间，N0和Nt分别代表对数生长期开始时间和对数生长期结束时间的细胞数。

#### 1.2.3 检测肺癌成球细胞的侵袭能力

通过Transwell实验检测肺癌成球细胞的侵袭能力。用Matrigel包被8 um孔径Transwell小室，37 ℃温育30 min使胶固化。在6孔板小孔内加入1mL DMEM/F12培养基（含10%血清和100 ng/mL IGF-1，bFGF，EGF），将小室放入备用。用含1%血清的DMEM/F12培养基调整待测细胞至3×10^5^/mL，在小室中加入600 μL该浓度的细胞悬液，37 ℃温育48 h后撤去小室，用专用棉签轻轻擦去Matrigel胶和上层细胞，避免损伤基底膜，再将上室浸泡于结晶紫染色液中30 min；然后取出上室在双蒸水中漂洗3次，晾干。用倒置荧光显微镜拍照（100×），计数穿过Matrigel生物胶落人小孔的细胞。

#### 1.2.4 检测肺癌成球细胞体内致瘤能力

将20只质量为18 g -20 g，8周-10周的雌性裸鼠（SPF级，由北京肿瘤研究所实验动物中心提供），随机分为两组。分别将100 μL（细胞数量分别为10^6^，5×10^5^，1×10^5^，5×10^4^，10^4^个）L9981球体细胞与等体积materiel混合，接种于实验组裸鼠腋窝皮下，对照组裸鼠皮下接种相同浓度的普通血清贴壁培养的L9981细胞。每周观察一次肿瘤生长状况，当形成肿瘤的最大直径大于1 cm时将全部小鼠断颈处死，拍照后摘取皮下肿瘤，测量肿瘤大小。

### 1.3 统计学分析

应用SPSS 17.0进行统计学分析，计量结果以Mean±SD表示。两样本的比较均采用独立样本的*t*检验，*P* < 0.05为差异有统计学意义。

## 2 结果

### 2.1 无血清悬浮培养肺癌细胞L9981球体与含血清常规培养L9981细胞的形态学比较

在倒置相差显微镜低倍镜下，在含血清培养基的普通培养皿中培养的L9981细胞贴壁生长，呈现杆状、短梭形，细胞核呈圆形或椭圆形，偏心位，多伪足，细胞大小均匀（图 1A）。将L9981细胞接种在超低吸附皿中，并用含有生长因子的无血清培养基进行培养24 h后，细胞开始大量死亡。残留细胞继续培养48 h后，细胞开始成球，可见到若干个细胞球形成（图 1B）。细胞球呈圆形或椭圆形，球体大小不等。细胞球折光性较强，多数悬浮细胞球不透明。球内细胞间紧密连接，难以区分细胞间界限。有时可见两个或以上悬浮细胞球相互结合的现象（图 1C）。细胞球培养第5-7天时，镜下培养基内可见少量细条状物，部分连于悬浮细胞球上（图 1D）。并且成球率达到高峰期，细胞球体体积达到最大大。继续培养第8-10天时，细胞球中心开始坏死（图 1E），细胞球数目减少，且逐步变为单细胞悬浮状态，并逐渐死亡（图 1F）。与贴壁肿瘤细胞相比较，悬浮肿瘤细胞生长成球较慢，花费时间长，最少需要1周才能形成大量可见球体。

**1:**
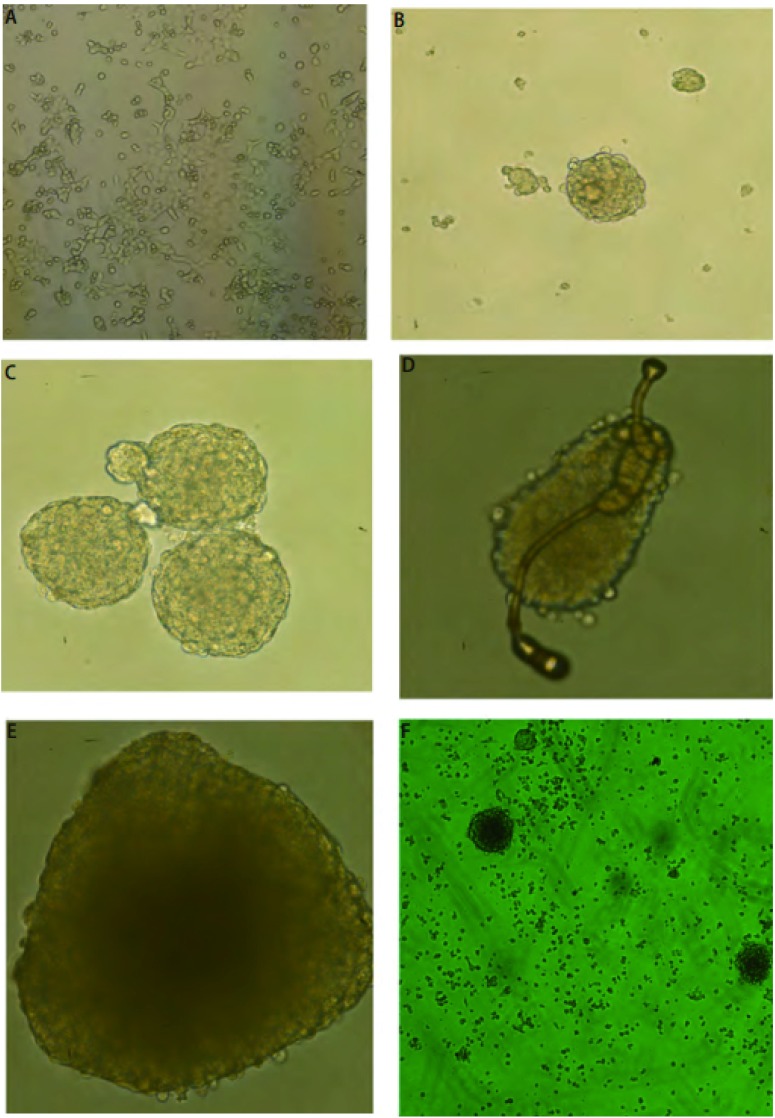
无血清悬浮培养L9981球体细胞和血清贴壁生长L9981细胞在显微镜下形态学差异。A：L9981含血清贴壁培养后形态（100×）；B：L9981无血清培养第3天形态（100×）；C：L9981无血清培养第5天形态（100×）D：L9981无血清培养第6天形态（200 ×）；E：L9981无血清培养第8天形态（100×）（细胞球坏死崩解）；F：L9981无血清培养第10天形态（100×）（细胞球坏死崩解成单细胞状态）。 Morphology of the L9981 spheres cells in serum-free suspension culture is different from the L9981 cells in serum adherent morphological under the microscope. A: the morphology of L9981 cell in serum adherent culture (100 ×); B: the morphol-ogy of L9981 cell in serum-free suspension on the third day (100 ×); C: the morphology of L9981 cell in serum-free suspension on the fifth day (100 ×); D: the morphology of L9981 cell in serum-free suspen-sion on the sixth day (200 ×); E: the morphology of L9981 cell in serum-free suspension on the eighth day (the spheres in necrosis and Collapse) (100 ×); F: the morphology of L9981 cell in serum-free sus-pension On the tenth day ((the spheres in single-cell state) (100 ×).

### 2.2 分别绘制无血清悬浮培养前后L9981细胞的生长曲线并计算倍增时间

无血清悬浮培养成球前后的L9981细胞的生长曲线均呈“S”型，符合肿瘤细胞无限细胞系的特征。当细胞处于对数生长期时，计算细胞倍增时间，在有血清培养条件下，成球的L9981细胞和贴壁的L9981的倍增时间分别为（33.00±1.44）h和（56.05±1.95）h（表 1，图 2），成球L9981细胞与血清贴壁培养L9981细胞相比生长更快。具有明显差异（*P* < 0.01）。实验重复3次（图 2），无血清悬浮培养后的细胞与血清贴壁生长的细胞相比，其增殖能力明显提高。

**1:** L9981球体细胞和含血清贴壁培养的L9981细胞倍增时间比较（Mean±SD，*n*=3） The comparation of Cell doubling time between L9981 spheres cells and L9981 in serum adherent culture

Cell culture conditions	Doubling time of cell growth	*P*(bilateral *P*-value)
L9981 spheres cells in serum –free culture	(33.00±1.44) h	< 0.001
L9981 in serum adherent culture	(56.05±1.95) h	

**2:**
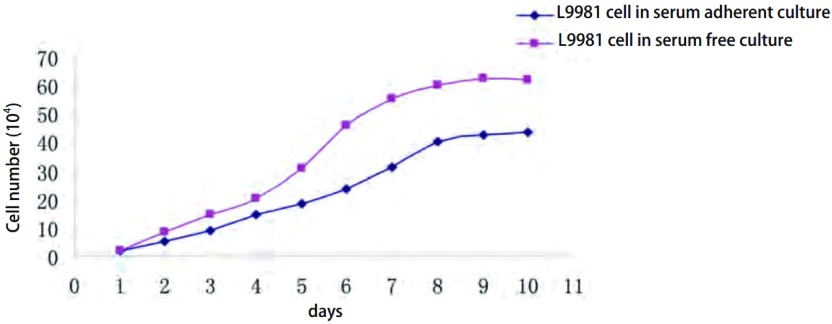
无血清悬浮培养L9981球体细胞与血清贴壁培养L9981细胞株的生长曲线比较（*P* < 0.05） Cell growth curve of L9981 spheres cells in serum-free suspension culture is compared with that of L9981 cell in serum adherent culture (*P* < 0.05)

### 2.3 Transwell实验结果

显微镜下随机计数5个视野内（100 ×）穿过基底膜的细胞数目，发现无血清悬浮培养后的L9981组平均视野中的侵袭细胞数目为282.75个（图 3A，表 2），而含血清贴壁培养的L9981细胞侵袭穿过基底膜的细胞数目为55.42个（图 3B，表 2）。L9981球体细胞在相同条件下穿过Matrigel胶进入下室的细胞数量是普通贴壁培养细胞的5倍以上，显示了无血清悬浮培养成球富集的细胞具有更强的侵袭性。

**3:**
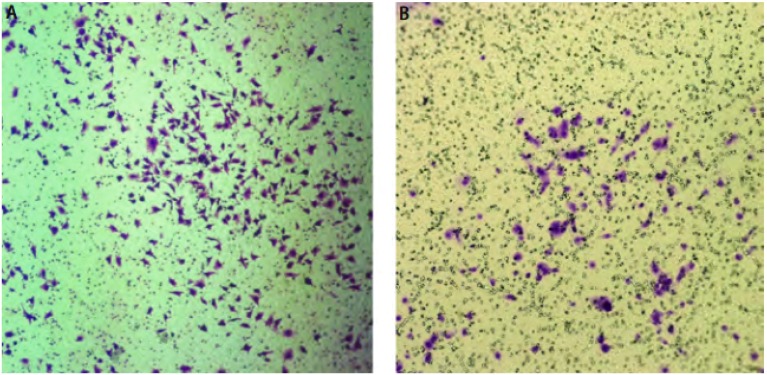
无血清悬浮培养L9981球体细胞株(A)与血清贴壁培养L9981(B)细胞株的transwell侵袭性比较（*P* < 0.05） The comparison of transwell chamber in-vasion between L9981 spheres cells in serum-free suspension culture (A) and L9981 in serum adherent culture(B)(*P* < 0.05)

**2:** L9981球体细胞和含血清贴壁培养的L9981细胞侵袭性比较（Mean±SD，*n*=3） The comparation of invasion between L9981 spheres cells and L9981 in serum adherent culture

Cell culture conditions	The average number through the Matrigel invasion of cells	*P*(bilateral *P*-value)
L9981 spheres cells in serum –free culture	282.75±1.27	*P* < 0.05
L9981 in serum adherent culture	55.42±1.42	

### 2.4 裸鼠体内成瘤实验结果

接种悬浮培养成球的L9981细胞的实验组裸鼠3周后开始成瘤，第6周处死时，共有5只裸鼠成瘤，成瘤率为5/10（表 3，图 4）。接种含血清贴壁培养L9981细胞的裸鼠始终均未见成瘤，成瘤率为0/10。无血清悬浮培养肺癌细胞球的体内成瘤能力显著高于血清培养贴壁肺癌细胞（*P* < 0.05）。

**3:** L9981球体细胞和含血清贴壁培养的L9981细胞的成瘤性比较 The comparison of transpntation tumor of mice between L9981 spheres cells and L9981 cells in serum adherent culture

Cell concentration injected in nude mice	L9981 spheres cells in serum –free culture	L9981ce l lsinserum adherent culture	Tumor size	*P*-value
2×10^6^（pre-test）	—	2/2	>1.0 cm×1.2 cm×1.0 cm	
1×10^6^	2/2	0/2	1.5 cm×2.0 cm×2.0 cm 1.1 cm×1.2 cm×1.4 cm	*P* < 0.05
5×10^5^	2/2	0/2	1.2 cm×1.4 cm×1.1 cm 0.8 cm×0.5 cm×1.0 cm	
1×10^5^	1/2	0/2	0.5 cm×1.3 cm×1.3 cm	
5×10^4^	0/2	0/2	0.0 cm×0.0 cm×0.0 cm	
10^4^	0/2	0/2	0.0 cm×0.0 cm ×0.0 cm	
Note: 0/2 behind the number 2 illustrates the nude mice numbers injected, the 0 is the numbers of nude mice with transpntation tumor. "-" illustrates the magnitude of non-inoculated mice (*P* < 0.05).				

**4:**
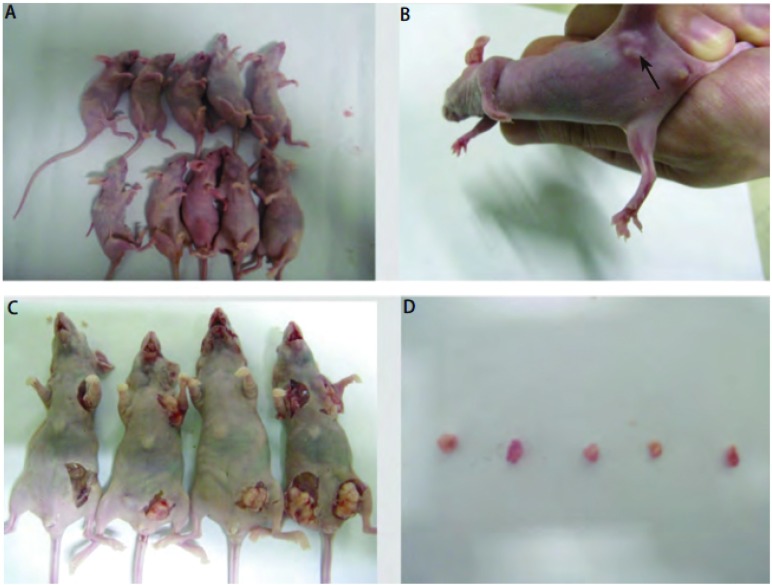
裸鼠成瘤情况。A：B组裸鼠接种血清贴壁培养细胞；B：A组活体裸鼠成瘤后均未成瘤可见皮下肿瘤；C：A组裸鼠体内产生肿瘤的摘取；D：摘取后肿瘤的形态和大小。 Transpntation tumor of mice. A: No tumor formation is observed in B groups of nude mice inoculated with L9981 cells in in serum adherent culture; B: A group of nude mice subcutaneous tumor is observed; C: the removal of the tumor generated in A group of nude mice; D: Morphology of the Transpntation tumor of mice is observed and tumor size is measured.

## 3 讨论

肿瘤干细胞理论为更好地治疗肿瘤带来了曙光^[7-10]^。肺癌是世界上死亡率最高的恶性肿瘤，迫切需要研究肺癌干细胞的生物学功能，为肺癌的治疗奠定理论基础。当前肺癌干细胞研究的焦点是分离和鉴定肺癌干细胞。一般方法是采用流式细胞仪分选可能的肺癌细胞亚群，再进行生物学鉴定，以证明其干细胞特性。应用流式细胞仪分选方法，Eramo等^[6]^提出CD133是一个有效的肺癌干细胞分选标记物，Ho等^[11]^利用SP（side popμlation）法筛选出具有部分干细胞特性肺癌细胞。近几年陆续发现ALDH、ALDH1、ALDH3A1、ASCL1和ALDH1A1可能是肺癌干细胞的筛选标记^[12-19]^。Leung等^[20]^发现NSCLC表达CD44阳性群（CD44^+^）肺癌细胞可能富含肺癌干细胞。Gutova等^[21]^发现SCLC中尿激酶纤溶酶原激活物受体阳性（uPAR+）细胞群可能含有肺癌干细胞，Koch等^[22]^发现PODXL-1（足糖萼蛋白样蛋白1）和Bmi1是SCLC中可能的肺癌干细胞分选标记。也有研究^[23]^显示CD133(+)/nes-tin(+)是可能的肺癌干细胞标记。但是，以上发现的可能肺癌干细胞筛选标记均存在争议。Meng的研究^[24, 25]^提出仅表达CD133尚不能作为肺癌干细胞的标记物。Cui等^[26]^研究认为CD133仅仅是SCLC中肺癌干细胞的一个临时标记，而并不能作为非NSCLC中肺癌干细胞的标记。

上述研究均是借鉴已知的肿瘤干细胞标记物来分离出一个肺癌细胞亚群，再鉴定其是否具有干细胞特性。但该方法存在明显不足之处，此方法借鉴其他肿瘤干细胞或正常肺干细胞的筛选标记来分选肺癌干细胞，分离出来细胞亚群，可能只是一部分类似祖细胞的细胞群——处于肺癌细胞发育过程中的比较靠近肿瘤细胞起源处的细胞，并没有筛选到真正的肺癌干细胞。理论上只有分离得到的单个的能致瘤的肺癌细胞才是真正的肺癌干细胞。目前利用已有的各肿瘤干细胞标记筛选出来的细胞都不能满足这个要求。同时，虽然这种分选方法是当前研究肿瘤干细胞的主流方法，但是一旦选取错误的分选标记进行分选，后续再验证这些筛选出的错误细胞亚群将毫无意义。整个分选过程中实验步骤繁琐，需要培养大量细胞，造成大量资源的浪费。在干细胞分选标记没有明确的情况下，这种分选方法并不足取。

我们依据肿瘤干细胞具有能在无血清悬浮培养条件下富集的特性，采用添加生长因子的无血清培养基对L9981进行悬浮培养，获得肺癌细胞球体。然后对分离到的肺癌细胞球体进行生物学功能鉴定，并鉴定分选到的细胞亚群是否是具有肺癌干细胞特性的细胞。此方法无需大量费用和复杂的细胞分离纯化过程。

癌细胞起源于上皮细胞，成贴壁生长，一旦阻止其贴壁，癌细胞将很快凋亡。而在无血清条件下，加入EGF，bFGF等细胞生长因子可以诱导肿瘤干细胞悬浮生长成球体。无血清悬浮培养作为一种体外富集干细胞的方法，最早见于神经干细胞的培养^[27]^，Ponti等^[8]^悬浮培养原代人乳腺癌细胞和MCF-7细胞株，发现它们均可在无血清条件下非粘附生长，形成乳腺癌细胞球，且可体外连续扩增传代，具有干细胞特性。Dontu等^[28, 29]^在超低吸附培养皿中，采用含EGF和B27生长因子的无血清培养基，直接诱导正常乳腺干细胞及乳腺癌干细胞形成球体。也有研究者^[30, 31]^通过此方法培养乳腺癌细胞以获得稳定的乳腺癌干细胞/祖细胞群。在添加生长因子的无血清培养条件下，细胞悬浮生长形成“球状”结构，从而限制了细胞的分化，可以达到富集干细胞的目的，并且球体细胞可以进行传代培养。

肿瘤细胞对无血清培养条件有一个适应过程，这一过程本身就是一种筛选。在缺少血清的环境中，相对分化成熟的细胞由于耐受不了长期的悬浮生长而凋亡，而相对未分化的细胞则存活下来并开始增殖。必须保证悬浮成球培养在超低吸附环境中进行，因为在无血清普通培养皿培养肿瘤细胞仍然不同程度的贴壁生长不能获得球体。作为一种初步分离、扩增干细胞的体外培养方法，无血清培养无疑具有简便、快速、实用性强的优点，可以获得足够数量的细胞用于后续的实验检测。相对于含血清培养而言，无血清悬浮培养基具有更高的成分限定性、各批产品之间更高的产品质量一致性、简化提纯和下游生产过程、便于控制培养的生理环境等优点。最新文献报道^[30, 32]^显示，采取低血清浓度进行干细胞的筛选取得了成功。

细胞对无血清培养条件有着较为严格的要求。为了使细胞适应无血清培养，必须保证使种入的细胞处于对数生长中期，活细胞率大于90%，细胞初次以较高的浓度接种。同时必须加入EGF和bFGF等生长因子，才能诱导细胞进入增殖周期大量扩增。缺乏这些因子则细胞长期处于该培养条件下不增殖或增殖缓慢。本研究采用的是2×10^4^起始浓度种入无血清低吸附培养皿中，与Eramo的实验相比，我们额外加入了IGF-1，产生的球体更多更明显。

肿瘤细胞动物成瘤实验是检验肿瘤干细胞的“金标准。在本研究中，在10%血清培养基中接种相同浓度的细胞时，L9981球体细胞比血清贴壁培养的L9981细胞生长更加迅速，且细胞倍增时间明显缩短（*P* < 0.05）。Transwell实验显示L9981球体细胞在相同条件下穿过Matri-gel胶进入下层的细胞数量是普通贴壁培养细胞的5倍以上。L9981球体细胞的侵袭能力显著高于普通血清贴壁培养的L9981细胞（*P* < 0.05），致瘤性实验比较显示仅1×10^5^个L9981球体细胞即可成瘤，而2×10^6^个普通血清贴壁培养的L9981细胞才能成瘤，两者相差20倍，具有明显差异（*P* < 0.05）。这些结果提示L9981球体细胞中确实富集了肺癌干细胞，也提示无血清悬浮培养只是富集了含干细胞/祖细胞的一个异质群，如果全部L9981球体细胞都是肺癌干细胞，那它们和普通肿瘤细胞相比成瘤性差异会更大。

总之，无血清悬浮培养作为一种富集干细胞的方法，该法得到的细胞仍然只是一个富含干细胞或祖细胞在内的异质性的细胞。肺癌干细胞处于肺癌肿瘤细胞发育的源头部位且单个肺癌干细胞就具有很强的致瘤能力。本研究虽然证实了人大细胞肺癌细胞株L9981中存在肺癌干细胞或者肺癌干细胞样的细胞。但是也迫切需要后续的实验检测这些球体细胞表面蛋白标记的表达，最终明确真正的筛选标记以分离肺癌干细胞。到底哪一个标记适合用于筛选肺癌干细胞，这需要我们进一步研究。
